# In Vitro Studies on Mg-Zn-Sn-Based Alloys Developed as a New Kind of Biodegradable Metal

**DOI:** 10.3390/ma14071606

**Published:** 2021-03-25

**Authors:** Yafeng Wen, Qingshan Liu, Weikang Zhao, Qiming Yang, Jingfeng Wang, Dianming Jiang

**Affiliations:** 1 Department of Orthopaedics, The First Affiliated Hospital of Chongqing Medical University, No. 1 Youyi Road, Yuzhong District, Chongqing 400016, China; wenyafeng@stu.cqmu.edu.cn (Y.W.); weikang-zhao@cqu.edu.cn (W.Z.); 2018010024@stu.cqmu.edu.cn (Q.Y.); 2 National Engineering Research Center for Magnesium Alloys, College of Materials Science and Engineering, Chongqing University, No. 174, Shapingba Main Street, Shapingba District, Chongqing 400044, China; 201709021005@cqu.edu.cn (Q.L.); jfwang@cqu.edu.cn (J.W.); 3 Department of Orthopaedics, The Third Affiliated Hospital of Chongqing Medical University, No. 1 Shuanghu Road, Yubei District, Chongqing 401120, China

**Keywords:** Mg-Zn-Sn alloy, corrosion behavior, mechanical properties, biocompatibility, osteoinductive activity

## Abstract

Mg-Zn-Sn-based alloys are widely used in the industrial field because of their low-cost, high-strength and heat-resistant characteristics. However, their application in the biomedical field has been rarely reported. In the present study, biodegradable Mg-1Zn-1Sn and Mg-1Zn-1Sn-0.2Sr alloys were fabricated. Their microstructure, surface characteristics, mechanical properties and bio-corrosion properties were carried out using an optical microscope (OM), X-ray diffraction (XRD), electron microscopy (SEM), mechanical testing, electrochemical and immersion test. The cell viability and morphology were studied by cell counting kit-8 (CCK-8) assay, live/dead cell assay, confocal laser scanning microscopy (CLSM) and SEM. The osteogenic activity was systematically investigated by alkaline phosphatase (ALP) assay, Alizarin Red S (ARS) staining, immunofluorescence staining and quantitative real time-polymerase chain reaction (qRT-PCR). The results showed that a small amount of strontium (Sr) (0.2 wt.%) significantly enhanced the corrosion resistance of the Mg-1Zn-1Sn alloy by grain refinement and decreasing the corrosion current density. Meanwhile, the mechanical properties were also improved via the second phase strengthening. Both Mg-1Zn-1Sn and Mg-1Zn-1Sn-0.2Sr alloys showed excellent biocompatibility, significantly promoted cell proliferation, adhesion and spreading. Particularly, significant increases in ALP activity, ARS staining, type I collagen (COL-I) expression as well as the expressions of three osteogenesis-related genes (runt-related transcription factor 2 (Runx2), osteopontin (OPN), and osteocalcin (Bglap)) were observed for the Mg-1Zn-1Sn-0.2Sr group. In summary, this study demonstrated that Mg-Zn-Sn-based alloy has great application potential in orthopedics and Sr is an ideal alloying element of Mg-Zn-Sn-based alloy, which optimizes its corrosion resistance, mechanical properties and osteoinductive activity.

## 1. Introduction

Biodegradable implants represented by magnesium (Mg) alloys have attracted increasing interest in the last few years. Compared with traditional metal materials, the biggest advantage of Mg alloys is the ability to be completely degraded gradually after exerting biological functions in the body, thereby avoiding subsequent surgical removal procedures [1,2]. Consequently, lifelong problems caused by permanent implants such as tissue dysfunction, long-term foreign body stimulation and local chronic inflammatory reactions can be effectively alleviated or eliminated [3]. In addition, the density and elastic modulus of Mg alloys are close to those of human cortical bone [4], which are more suitable for orthopedic implant applications and can effectively eliminate/decrease the stress shielding effect and resulting osteoporosis induced by traditional metal materials [5]. Moreover, Mg implants have been proven to stimulate the formation of new bone [6].

However, the rapid degradation rate of Mg alloys in the physiological environment is the main reason hindering its clinical application. Furthermore, during the degradation of Mg alloys, it will result in increasing of local pH and accumulation of hydrogen (H_2_), causing inflammation and destruction of surrounding tissues [7]. This also means that the Mg alloy may lose sufficient mechanical support strength before the expected task is completed. Therefore, the development of new biodegradable Mg alloy by adding new alloy elements is a current research focus.

From a clinical viewpoint, an ideal Mg alloy orthopedic implant should meet the following standards: First, the degradation rate should be less than or equal to 0.5 mm/y. Second, the mechanical strength must be higher than 200 MPa and the elongation is preferably greater than 10%. Simultaneously, the mechanical integrity should be maintained for at least 90–180 days in vivo [6,8]. Third, the limit of acceptable H_2_ evolution rate for humans was reported as 0.01 mL/cm^2^/day [6,9]. Moreover, biosafety and bioactivity, etc. must also be considered. To date, Mg-based alloys that have been developed and intensively studied include Mg-Zn [10], Mg-Ca [11], Mg-Zr [12], Mg-Sr [13] et al., and ternary or multicomponent alloys developed on those bases. In 2013, magnesium-based alloy compression screws received the CE mark and became the first Class III medical device made of Mg alloy approved for clinical use. However, due to the presence of rare-earth (RE) metal elements, their long-term biosafety remains controversial.

Mg-Zn-Sn-based alloys are widely used in the industrial field due to their low-cost, high-strength and heat-resistant characteristics. However, there are few reports on application of Mg-Zn-Sn-based alloys in the biomedical field. Tin (Sn) is an ultra-trace element that does not exceed 1 mg per kilogram of body weight in the human body. The task of Sn as a trace element in the body is not really known, but a deficiency of Sn may disrupt kidney function. Studies have reported that Sn may be a good alloying element for biodegradable magnesium alloys [14]. In addition, Sn is an element with high H_2_ evolution overpotential, which can control H_2_ release of Mg alloys [15]. Strontium (Sr), as an essential trace element for the human body, has been in good repute to endorse the proliferation of osetoblasts and restrain the activity of osteoclasts [16]. Moreover, microalloying Sr in different Mg-based systems can indeed improve the performance of Mg alloys [17], but in the Mg-Zn-Sn-based alloy, no relevant literature has been reported.

In the present study, the application possibility and potential in the biomedical field of Mg-1Zn-1Sn and Mg-1Zn-1Sn-0.2Sr alloys were systematically studied, focusing on the surface characteristics, mechanical properties, corrosion performance and in vitro biocompatibility and bioactivity.

## 2. Materials and Methods

### 2.1. Alloys Preparation

The ingots of Mg-1Zn-1Sn and Mg-1Zn-1Sn-0.2Sr alloys were prepared by casting from melting Pure Mg (purity > 99.98), pure Zn (purity > 99.99), pure Sn (purity > 99.99) and Mg-15 wt.% Sr master alloys in an electric resistance furnace under the protection of an SF_6_ and CO_2_ gas mixture in a graphite crucible. 

The actual compositions of the alloys are shown in Table 1. After homogenization treatment at 500 °C for 6 h, the ingot casting was extruded at 280 °C with an extrusion ratio of 28:1. Subsequently, the Mg–1Zn–1Sn and Mg–1Zn–1Sn-0.2Sr ingots were cut into sheets, and commercial pure-Mg (p-Mg) was used as a reference. The sheets were cut into φ15 mm × 1 mm wafers for follow-up experiments.

### 2.2. Microstructure Analysis and Mechanical Testing

The microstructure and surface morphology of the samples were examined by using an OM (OLYMPUS, Fuji, Japan) and a SEM (Vega III LMH, TESCAN, Shanghai, China) equipped with energy-dispersive X-ray spectroscopy (EDS). Moreover, the phase compositions were detected by XRD (Rigaku, Tokyo, Japan) with CuKα radiation at a scan rate of 5°/min. Compression tests were conducted in accordance with the procedures listed in ASTM standard E9-09 [18] by using a universal testing machine (AOUDE, Beijing, China), and the initial strain rate is 10^−3^/s. According to the specifications of American Society for Testing Materials (ASTM) standard B557-15 [19], tensile specimens were tested at room temperature, and the tensile speed is 2 mm/min. Three parallel samples were tested for each group of materials.

### 2.3. In Vitro Corrosion Evaluation

Potentiodynamic polarization (PDP) curves were obtained using an electrochemical workstation (CHI600C, CHINSTRUMENTS, Shanghai, China) in simulated body fluid (SBF). Specific details are given elsewhere [20]. Briefly, The Mg-1Zn-1Sn and Mg-1Zn-1Sn-0.2Sr alloys were embedded in epoxy resin as the working electrode (only 1 cm^2^ exposed). Platinum foil was used as the counter electrode, and a saturated calomel electrode was employed as reference. Afterward, a PDP curve was performed at a scanning speed of 1 mV·s^−1^ for all the measurements. Taking ASTM G102-89 [21] as the guide standard, the Tafel method was used to calculate the values of corrosion current density (i_corr_) and corrosion potential (E_corr_).

Immersion tests were carried out in SBF. The chemical compositions of SBF were shown in Table 2. The ratio of SBF volume to the surface area of the material was 50 mL: 1 cm^2^. The H_2_ evolution and pH value were monitored during a period of 336 h. The mass loss was also recorded after 3, 7, 15 and 35 day-immersion, respectively. A mixed solution composed of chromic acid (180 g/L) and silver nitrate (10 g/L) was used to eliminate the corrosion products on the surface of samples. The corrosion rate (CR) was calculated by the following formula:

CR = (K × W) ÷ (D × T × A)

where the coefficient K = 8.76 × 10^4^, W is the mass loss (g), D is the density of the material (g.cm^−3^), T is the exposure time (h) and A is the sample area exposed to the solution (cm^2^). 

### 2.4. In Vitro Cell Test

#### 2.4.1. Cell Culture and Preparation of Extraction

The murine calvarial preosteoblasts (MC3T3-E1) were utilized to realize the evaluation of in vitro experiments. P-Mg extracts and normal culture medium were used as a control and reference, respectively. The extraction was prepared according to a reference [22]. In short, samples were immersed in Dulbecco’s modified Eagle’s medium (DMEM, Hyclone) containing 10% (*v*/*v*) fetal bovine serum (FBS, Gibco), 100 U/mL penicillin and 100 mg/mL streptomycin for 72 h under a standard cell culture environment (95% humidity, 5% CO_2_ and 37 °C). The extraction ratio of medium volume to material weight was 5 mL/g.

#### 2.4.2. Cytocompatibility and Cell Morphology

MC3T3-E1 cells were seeded in 96-well plates with a density of 1 × 10^4^ cells per well and incubated for 1 day to allow the cells to adhere completely, and then replace the culture medium with the extracts. After incubating for 1, 2 and 3 days, 20 μL CCK-8 assay reagent was added to each well and incubated for another 1 h in an incubator. The optical density (OD) was measured by using a microplate reader (Thermo Scientific, Waltham, MA, USA) at the wavelength of 450 nm.

The live/dead cell assay was performed according to the protocol from the manufacturer (BestBio, Shanghai, China). In brief, cells treated with extracts for 3 days were stained with 200 μL (1:10,000) of calcein-AM solution for 30 min and 200 μL (1:5000) of PI solution for 5 min. Images were collected by fluorescence microscopy (Zeiss, Jenoptik, Germany); the viable cells were stained green, while dead cells were stained red.

The morphology of cells co-cultured with extracts and materials were observed by CLSM. Briefly, cells were fixed and permeabilized with 4% paraformaldehyde (PFA) and 0.1% Triton X-100 successively, and then dyed the nucleus with 4′,6-diamidino-2-phenylindole dilactate (DAPI). F-actins were stained with Actin-Tracker Green (Beyotime Biotechnology, Shanghai, China) and rhodamine-phalloidin (Sigma-Aldrich Co., Shanghai, China). Fluorescence images were captured under the same exposure condition.

SEM was used to observe the cell adhesion and spreading morphology on the surface of the samples. In brief, samples were immersed in 2.5% glutaraldehyde for 15 min. Next, samples were dehydrated in graded ethanol (10, 30, 50, 70, 90, and 100% ethanol sequentially; 10 min each) and then immersed in graded tertiary butanol (50, 70, 90, 95, 100 and 100% tertiary butanol sequentially, 5 min each). Finally, samples were air-dried and sprayed with gold. SEM was used to observe the morphology of cells.

#### 2.4.3. In Vitro Osteogenic Differentiation

The alkaline phosphatase (ALP) assay was used to analyze the intracellular ALP activity. Briefly, cells seeded in 12-well plates were incubated with extracts (containing 100 nM dexamethasone, 10 mM β-glycerophosphate, 50 mM ascorbate and glutamine.) for 14 days and the extracts were replaced every other day. The cellular ALP expression was detected by BCIP/NBT ALP Color Development Kit (Beyotime, Shanghai, China) and corresponding quantitative analysis was measured by the Alkaline Phosphatase Assay Kit (Beyotime, Shanghai, China) according to manufacturer’s instructions.

Calcium nodules, an important sign of extracellular matrix mineralization, was qualitatively tested by Alizarin Red S (ARS) (Sigma-Aldrich Co., Shanghai, China) staining. In brief, after 14 days of osteogenesis induction, samples were collected and fixed with 4% PFA for 30 min and then stained with 1% AR solution for 45 min. Then, the calcium nodules were observed under the microscope. In order to semi-quantitatively analyze the mineralization of extracellular matrix, cetylpyridinium chloride (10%, Sigma-Aldrich Co., Shanghai, China) solution was used to dissolve the mineralized nodules, and then the absorbance value of the dissolving solution was detected at 620 nm by using a microplate reader (Thermo Scientific, Waltham, MA, USA).

The expression of COL-I protein after 14 days osteogenic induction was determined by immunofluorescence staining. Cells were washed gently with PBS three times and then fixed in 4% PFA for 30 min. Then, Triton X-100 (0.1 *v/v*%) and bovine serum albumin (10%) were used to permeabilize and block cells, respectively. Next, incubated the cells overnight with anti-COL-I (Abcam) primary antibody. Afterward, cells were incubated with fluorescent secondary antibody for 1 h. Finally, cells were stained with DAPI for 5 min and observed by CLSM.

The expression of osteogenic genes such as Runx2, OPN, and Bglap were determined by qRT-PCR. Briefly, MC3T3-E1 cells were harvested for RNA extraction using Trizol (Sigma-Aldrich Co., Shanghai, China) method. Nanodrop 2000 (Thermo Scientific, Waltham, MA, USA) was used to detect the purity and concentration of RNA. The First Strand cDNA Kit (Takara, Dalian, China) was employed to reverse transcribe the total mRNA into cDNA. Next, 1 µL of synthesized cDNA was taken from each group and added into a 10 μL reaction system containing SYBR Green Mastermix and primers (Table 3). The expression of Runx2, CON and Bglap was quantified by the ABI 7900HT real-time PCR system (Applied Biosystems, Foster City, California, USA), β-actin was employed as a housekeeping gene. The expression of relative genes was calculated by using the 2^−△△CT^ formula.

### 2.5. Statistical Analysis

Statistical analysis was performed using a Student’s *t*-test for two groups comparison and one-way ANOVA followed by post-hoc tests for multiple-group comparisons via SPSS 18.0 software. Results that were statistically significant were determined by *p*-values < 0.05. 

## 3. Results and Discussion

### 3.1. Microstructures and Electrochemical Evaluations

**Figure 1:**
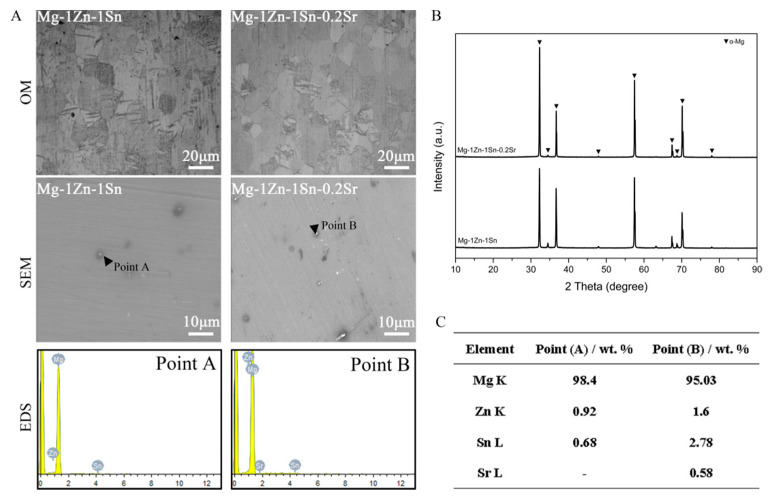
(**A**) Optical microstructure, SEM images, and corresponding EDS results of as-extruded Mg-1Zn-1Sn and Mg-1Zn-1Sn-0.2Sr alloys. The black triangle indicates the second phase. (**B**) XRD patterns of Mg-1Zn-1Sn and Mg-1Zn-1Sn-0.2Sr. (**C**) The chemical composition of point A and point B in Figure 1A.

Figure 1A shows the OM, SEM images and corresponding EDS diagram of the Mg-1Zn-1Sn and Mg-1Zn-1Sn-0.2Sr alloys. From the OM images, the grain size was slightly reduced from 26 ± 3 μm to 20 ± 2 μm with the incorporation of Sr, which is consistent with a previous study indicating that Sr has the effect of grain refinement [23,24,25]. With the addition of Sr, the second phase (bright spot) in the alloy matrix increases, the volume fraction of the second phase increased from 0.5% (Mg-1Zn-1Sn) to 1.1% (Mg-1Zn-1Sn-0.2Sr). To determine the composition of the bright spot, EDS was used to analyze the two marked points (A and B) in the alloys. The results (Figure 1C) showed that the elemental composition of point A included Mg, Zn and Sn, while those of point B included Mg, Zn, Sn and Sr, which was the same as that of the alloy matrix.

**Figure 2:**
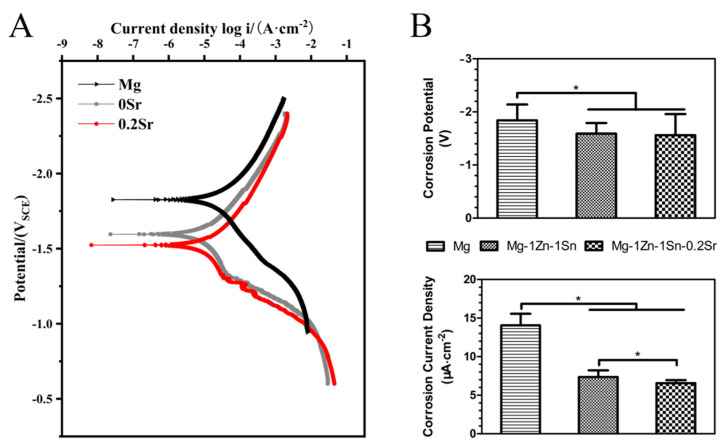
(**A**) Representative PDP curves of as-extruded Mg-1Zn-1Sn and Mg-1Zn-1Sn-0.2Sr alloys, (**B**) corrosion potential (E_corr_) and corrosion current density (i_corr_) obtained from Tafel extrapolation of PDP curves; values are mean ± SD, n = 3, * *p* < 0.05.

In order to further analyze the phase composition of the alloy microstructure, the XRD patterns of the Mg-1Zn-1Sn and Mg-1Zn-1Sn-0.2Sr alloys are shown in Figure 1B. The microstructure of both alloys is mainly composed of α-Mg; although the second phase can be clearly observed in the SEM images, it is difficult to detect by XRD due to the low content and high solid solubility of Zn and Sn in Mg. Moreover, the content of Sr is as low as 0.2 wt.%, which may even exceed the detection accuracy of XRD. Figure 2A shows the representative dynamic polarization (PDP) curves of p-Mg, Mg-1Zn-1Sn and Mg-1Zn-1Sn-0.2Sr samples, and the values of E_corr_ and I_corr_ are also obtained (Figure 2B). Significant differences in I_corr_ values were detected between the mean value for Mg-1Zn-1Sn (7.36 ± 0.86 μA cm^−2^) and Mg-1Zn-1Sn-0.2Sr (6.55 ± 0.41 μA cm^−2^), both of which were all significantly lower than p-Mg (14.05 ± 1.5). Similarly, the E_corr_ of p-Mg was significantly greater than Mg-1Zn-1Sn and Mg-1Zn-1Sn-0.2Sr. Electrochemical test results confirmed that the corrosion resistance of Mg-1Zn-1Sn and Mg-1Zn-1Sn-0.2Sr was significantly better than p-Mg. Moreover, the addition of Sr can further reduce the I_corr_ of Mg-1Zn-1Sn alloy to enhance corrosion resistance.

### 3.2. Mechanical Properties

**Figure 3:**
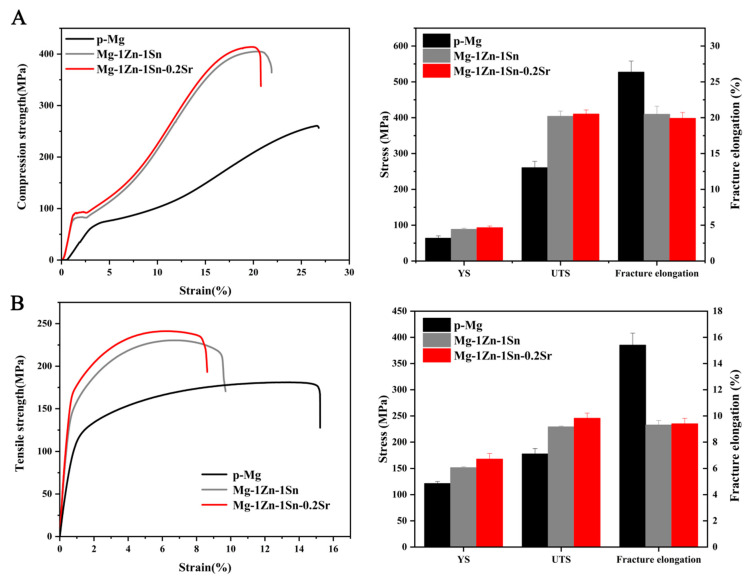
Mechanical properties of the p-Mg, Mg-1Zn-1Sn and Mg-1Zn-1Sn-0.2Sr alloys: (**A**) Compression stress–strain curves and graphs; (**B**) tensile stress–strain curves and graphs.

The mechanical properties of Mg-1Zn-1Sn and Mg-1Zn-1Sn-0.2Sr alloys are shown in Figure 3. The compressive yield strength, ultimate compressive strength, and compressive strain of p-Mg were 64 ± 6 MPa, 261 ± 18 MPa and 26.4 ± 1.5%, those of Mg-1Zn-1Sn alloy were 88 ± 3 MPa, 404 ± 14 MPa and 20.5 ± 1.1%, and those of Mg-1Zn-1Sn-0.2Sr alloy were 93 ± 5 MPa, 410 ± 12 MPa and 19.9 ± 0.8%, respectively. The tensile yield strength, ultimate tensile strength, and tensile strain of p-Mg were 121 ± 3 MPa, 178 ± 10 MPa and 15.4 ± 1%, those of Mg-1Zn-1Sn alloy were 151 ± 2 MPa, 229 ± 1 MPa and 9.0 ± 0.9%, and those of Mg-1Zn-1Sn-0.2Sr alloy were 168 ± 8 MPa, 245 ± 8 MPa and 9.0 ± 0.4%, respectively. The mechanical strength of Mg-1Zn-1Sn and Mg-1Zn-1Sn-0.2Sr were significantly stronger than that of p-Mg. It also showed that the addition of Sr further improved the mechanical properties of Mg-1Zn-1Sn alloy. We speculate that the reasons for the improved mechanical properties may be due to the second phase strengthening [26,27] or slight texture variations, and the specific mechanism still needs further study.

### 3.3. Immersion Tests

**Figure 4:**
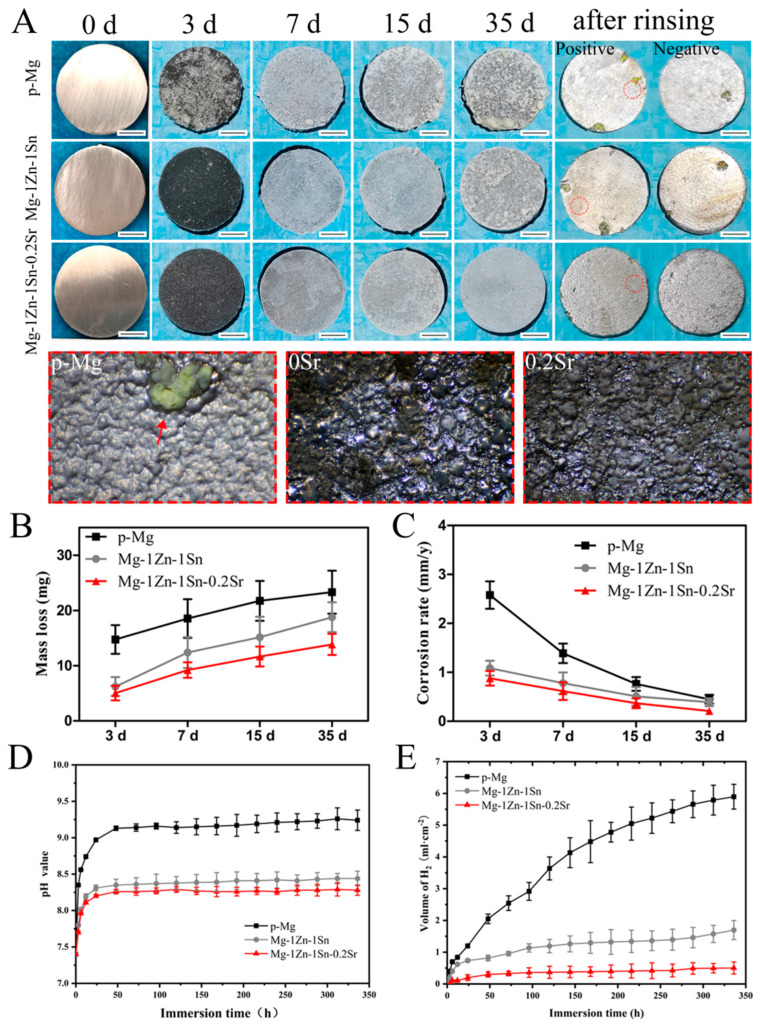
(**A**) Macroscopic images of p-Mg, Mg-1Zn-1Sn and Mg-1Zn-1Sn-0.2Sr at each prescribed time point during 35 days of immersion degradation in SBF. All wafers had a starting dimension of 15 mm in diameter and 1 mm in thickness. The red arrow indicates the typical pitting characteristics of p-Mg. (**B**) Mass loss, (**C**) corrosion rate calculated by mass loss, (**D**) pH value and (**E**) H_2_ release of p-Mg, Mg-1Zn-1Sn and Mg-1Zn-1Sn-0.2Sr at various immersion times in SBF. Scale bar = 5.

The results of immersion tests about general observation, H_2_ evolution, pH change, mass loss and corrosion rate calculated by mass loss are shown in Figure 4. Obviously, the degradation products were deposited on the surface of all samples as the extension of the immersion time. Specifically, corrosion products deposited on the surface of p-Mg samples were more and uneven, while those of that on the Mg-1Zn-1Sn and Mg-1Zn-1Sn-0.2Sr samples are less and evenly distributed. After removing the corrosion products, there are obvious corrosion holes on p-Mg and Mg-1Zn-1Sn samples, while the Mg-1Zn-1Sn-0.2Sr sample tends to be uniformly corroded without obvious corrosion pits. 

Mg degrades in physiological solutions according to the following reaction.

Mg_(s)_ + 2H_2_O_(aq)_ → Mg(OH)_2(s)_↓ + H_2(g)_↑

Mg(OH)_2(s)_ + 2Cl^−^_(aq)_ → MgCl_2(s)_ + 2OH^−^

During the first 24 h, the volume of H_2_ in Mg-1Zn-1Sn and Mg-1Zn-1Sn-0.2Sr samples increased rapidly, and then leveled off. It is obvious that the H_2_ release of Mg-1Zn-1Sn was much less than that of p-Mg, and when 0.2 wt.% Sr was added to the alloy, the amount of H_2_ evolution was further reduced. The total H_2_ released from Mg-1Zn-1Sn and Mg-1Zn-1Sn-0.2Sr samples over 336 h were 0.42 ± 0.02 mL·cm^−2^ and 0.13 ± 0.01 mL·cm^−2^, respectively, both of which were significantly lower than that of p-Mg samples (1.45 ± 0.41 mL·cm^−2^). We infer that the main reasons contain the following aspects. First, the Sn addition will decrease the H_2_ evolution of Mg alloys substantially for its high H_2_ evolution overpotential [16,28,29], which can effectively capture the H atom than the matrix and inhibit the H_2_ evolution rate [30]. Secondly, Sr can further moderate the corrosion rate of Mg alloys by refining the grains and decreasing the corrosion current density, thereby inhibiting H_2_ evolution [17,31].

The pH values of Mg-1Zn-1Sn group are comparable to that of Mg-1Zn-1Sn-0.2Sr group. In the first 24 h, the pH value of Mg-1Zn-1Sn group increased from 7.4 to 8.31, those of Mg-1Zn-1Sn-0.2Sr group increased from 7.4 to 8.25, and then maintained a relatively gentle upward trend throughout the inspection process. However, the pH value of Mg group increased rapidly from 7.4 to 8.9, which indicated that Mg-1Zn-1Sn and Mg-1Zn-1Sn-0.2Sr have better corrosion resistance than p-Mg.

Next, the corrosion rate based on mass loss during the immersion process was calculated, the mass loss of p-Mg, Mg-1Zn-1Sn and Mg-1Zn-1Sn-0.2Sr was 23.31 ± 3.91 mg, 18.78 ± 2.75 mg and 13.85 ± 1.93 mg after 35 days of immersion, and the corrosion rate was 0.44 ± 0.09, 0.31 ± 0.05 and 0.20 ± 0.03 mm/y, respectively. The average corrosion rate calculated from H_2_ evolution, mass loss and I_corr_ of p-Mg, Mg-1Zn-1Sn and Mg-1Zn-1Sn-0.2Sr alloys in SBF solution was shown in Table 4.

**Figure 5:**
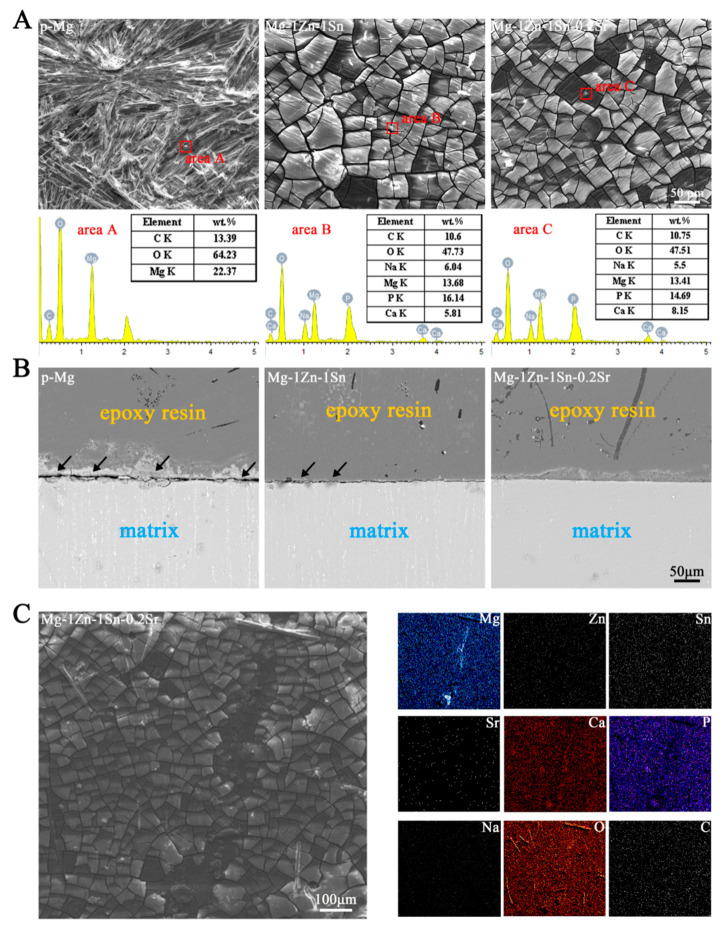
(**A**) Surface topographies and EDS results of p-Mg, Mg-1Zn-1Sn and Mg-1Zn-1Sn-0.2Sr alloys after 7 days of immersion in SBF; scale bar = 50 μm. (**B**) The morphologies of the cross-section of p-Mg, Mg-1Zn-1Sn and Mg-1Zn-1Sn-0.2Sr alloys after 7 days of immersion in SBF; scale bar = 50 μm. The black arrow indicates corrosion products. (**C**) SEM-EDS composite image of the surface of Mg-1Zn-1Sn-0.2Sr sample immersed in SBF at 37 °C for 7 days, scale bar = 100 μm.

Figure 5 shows the surface topographies, elemental compositions and morphologies of a cross-section of p-Mg, Mg-1Zn-1Sn and Mg-1Zn-1Sn-0.2Sr after immersion in SBF for 7 days. The corrosion products on the p-Mg samples had an uneven thickness and were in the form of crystal clusters, while those of that on the Mg-1Zn-1Sn and Mg-1Zn-1Sn-0.2Sr samples were uniform and dense with deposited white clusters/particles. The EDS results demonstrate that the corrosion products mainly compose of oxygen (O), magnesium (Mg), phosphorous (P) and calcium (Ca) on Mg-1Zn-1Sn and Mg-1Zn-1Sn-0.2Sr samples, while the corrosion products on p-Mg are mainly carbon (C), oxygen (O) and magnesium (Mg), indicating that Ca and P are more likely to be deposited on the surface of Mg-1Zn-1Sn and Mg-1Zn-1Sn-0.2Sr alloys, which are more conducive to the osteogenic differentiation of cells [32]. The formation of a large number of corrosion products was observed from the cross-sectional image of the p-Mg sample, indicating that the surface corrosion was serious, while the Mg-1Zn-1Sn sample showed fewer corrosion products, and the cross-section was clear and uniform. Remarkably, the cross-section of Mg-1Zn-1Sn-0.2Sr sample had the shallowest corrosion beneath the surface; no obvious corrosion products were observed.

### 3.4. Cell Viability, Cytocompatibility and Cell Morphology

**Figure 6:**
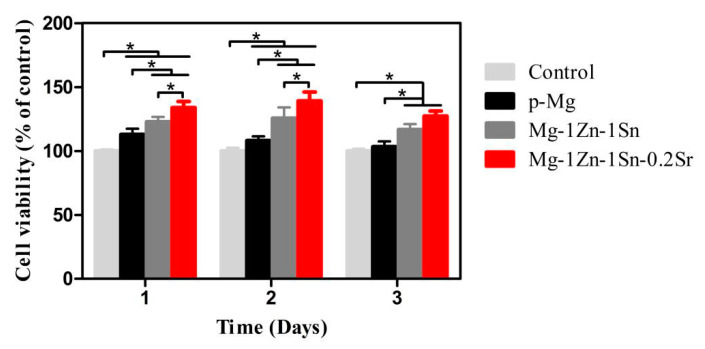
Cell viability of MC3T3-E1 cells cocultured with extracts for 1, 2 and 3 days. Values are mean ± SD, n = 3, * *p* < 0.05.

The viability of different extracts towards MC3T3-E1 is shown in Figure 6. The cell viability of Mg-1Zn-1Sn and Mg-1Zn-1Sn-0.2Sr reached the highest on the second day, which were 108 ± 3%, 125 ± 8% and 139 ± 6%, respectively, of the control group. This suggested that Mg-Zn-Sn-based alloys have excellent cell compatibility and can significantly promote cell proliferation.

**Figure 7:**
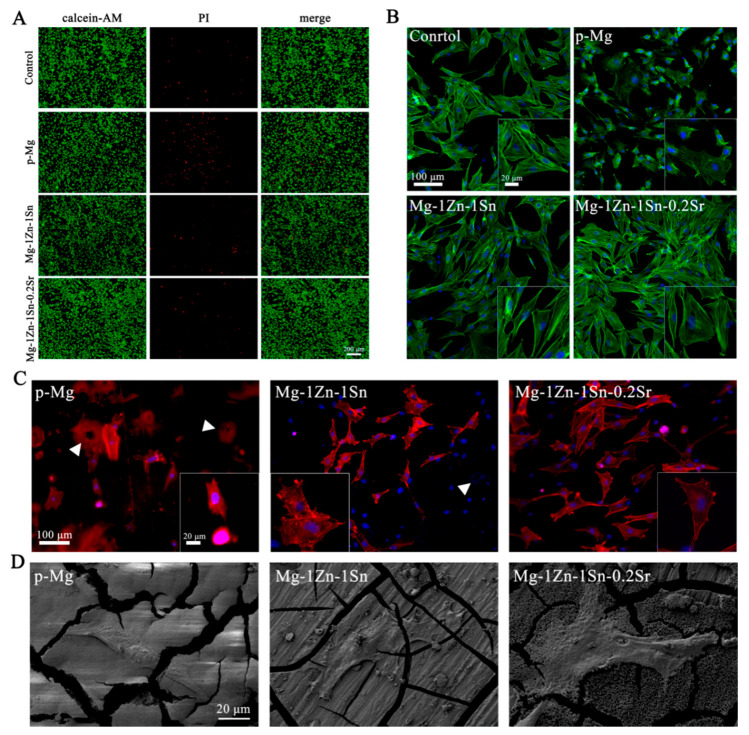
(**A**) Live/dead staining of MC3T3-E1 cells coculture with p-Mg, Mg-1Zn-1Sn and Mg-1Zn-1Sn-0.2Sr alloy extracts and DMEM control for 3 days; scale bar = 200 μm. (**B**) Actin-nucleus co-staining of MC3T3-E1 morphologies coculture with p-Mg, Mg-1Zn-1Sn and Mg-1Zn-1Sn-0.2Sr alloy extracts and DMEM control for 3 days. Scale bar = 100 μm. Insets were taken at 200 original magnification with scale bar = 20 μm. (**C**) Fluorescence images of MC3T3-E1 adhered to the surface of p-Mg, Mg-1Zn-1Sn and Mg-1Zn-1Sn-0.2Sr alloys after coculture for 3 days. Blue indicates nuclei, and red indicates cytoskeleton. White triangle indicates corrosion pit on the sample surface. The scale bar = 100 µm. Insets were taken at 200× original magnification with scale bar = 20 μm. (**D**) Morphologies of MC3T3-E1 cells adhered to the surface of p-Mg, Mg-1Zn-1Sn and Mg-1Zn-1Sn-0.2Sr alloys after coculture for 1 day. Scale bar = 20 μm.

Figure 7A shows the live/dead staining images of MCET3-E1 co-cultured with each extract for 3 days. Live cells with green fluorescence almost filled the entire field of view, while dead cells with red fluorescence also existed. The number of dead cells in the p-Mg group was obviously more than in other groups. However, no significant difference was detected among the control, Mg-1Zn-1Sn, and Mg-1Zn-1Sn-0.2Sr groups. These results also demonstrated the excellent cytocompatibility of the Mg-1Zn-1Sn and Mg-1Zn-1Sn-0.2Sr alloys.

Fluorescence images of MC3T3-E1 cultured in different extracts for 3 days are summarized in Figure 7B. Cells showed satisfactory adhesion state in multiple directions as well as intercellular connections and visibly stained cytoskeleton filaments in Mg-1Zn-1Sn and Mg-1Zn-1Sn-0.2Sr groups. Obviously, the F-actin area of cells adhered to the p-Mg samples was significantly smaller compared with the control, Mg-1Zn-1Sn and Mg-1Zn-1Sn-0.2Sr groups, which may be attributed to the rapid degradation of p-Mg in the early stage of immersion, resulting in drastic changes in the metal ion concentration, pH value and osmotic pressure, etc. [33].

Fluorescence and SEM images from a direct culture on the surfaces of the p-Mg, Mg-1Zn-1Sn and Mg-1Zn-1Sn-0.2Sr samples for one day are shown in Figure 7C,D. Attached cells were observed on all samples. The number of MC3T3-E1 on the surface of p-Mg was less and cannot spread well, which perform round or long fusiform morphologies. In contrast, cells on the surface of Mg-1Zn-1Sn and Mg-1Zn-1Sn-0.2Sr samples increased significantly and expanded well, which perform spindle or polygonal morphologies and have more pseudopods. 

**Figure 8:**
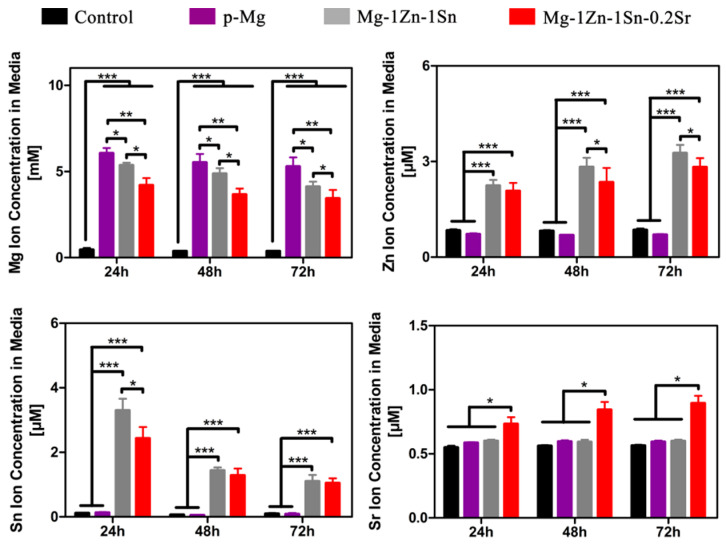
Mg, Zn, Sn and Sr ion concentrations in culture medium incubated with the samples during a 72 h period. n = 3 for all groups and time points. Values are mean ± SD. * *p* < 0.05, ** *p* < 0.01, *** *p* < 0.001.

In addition, corrosion holes were found on the surface of p-Mg and Mg-1Zn-1Sn samples—yet were not found on Mg-1Zn-1Sn-0.2Sr sample—indicating that the corrosion of the Mg-1Zn-1Sn-0.2Sr sample was more uniform and slower. Ion concentration in the extracts is also determined by inductively coupled plasma optical emission spectrometry (ICP-OES) (Figure 8). The Mg ions in the Mg-1Zn-1Sn and Mg-1Zn-1Sn-0.2Sr groups were lower than p-Mg group, implying their better corrosion resistance in vitro. Moreover, the solubilized Mg, Zn, Sn and Sr ions in the media were all within the average daily intake range of the human body [34,35], indicating their excellent biosafety.

Cell adhesion to the surface of the material is the most important step and is crucial for subsequent cell proliferation, long-term functions and organization of tissues [36]. In the present study, the adhesion of cells may be affected by many factors, such as ion concentration, pH value, material surface morphology and H_2_ evolution, etc. Romani A. et al. reported that the active transport of Mg^2+^ ions across the cell membrane was strictly controlled to keep the intracellular concentration within a normal range, regardless of the extracellular concentration [37]. In addition, the bicarbonate buffer system in the DMEM medium can effectively reduce the rapid increase in pH caused by the degradation of the material [20]. This indicates that the Mg^2+^ concentration and pH value in the medium are unlikely to be the main factors affecting cell adhesion. Therefore, we believe that changes in surface morphology and H_2_ evolution caused by substrate degradation may play important roles in regulating cell adhesion. 

As shown in Figure 4 and Figure 5, the faster the material corroded, the more severe the surface morphology changed, the more the H_2_ produced. The CLSM results (Figure 7C) indicated that cells on the surface of Mg-1Zn-1Sn and Mg-1Zn-1Sn-0.2Sr samples showed more adherence and better adhesion morphology (significantly larger F-actin area and more pseudopods), which was consistent with the results observed by SEM (Figure 7D). The above-mentioned results indicated that Mg-1Zn-1Sn and Mg-1Zn-1Sn-0.2Sr alloys were more suitable for cell adhesion and spreading. We think that the reasons may be as follows: As an element with high H_2_ evolution overpotential, Sn can effectively inhibit the H_2_ evolution of Mg alloys [28], thereby minimizing the adverse effect of H_2_ release on cell adhesion. Moreover, the incorporation of Sr further improves the corrosion resistance and enhances cell viability and proliferation.

### 3.5. In Vitro Osteogenesis Ability

**Figure 9:**
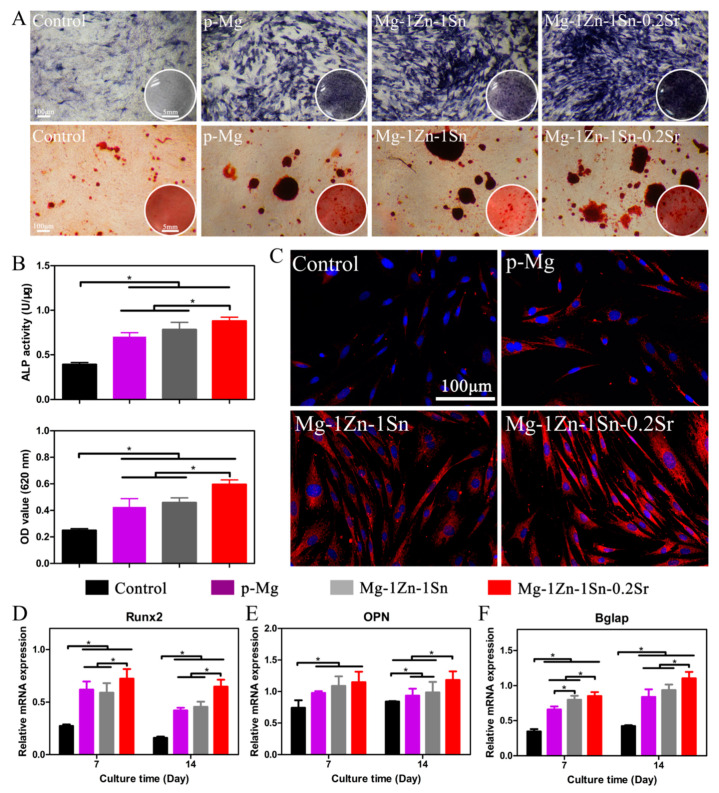
(**A**) Images of ALP activity and matrix mineralization, and (**B**) corresponding quantitative analysis. (**C**) Immunofluorescent staining of the expression of COL-I protein after osteogenic induction for 14 days. Osteogenesis-related genes Runx2 (**D**), OPN (**E**) and Bglap (**F**) expression in MC3T3-E1 cells after osteogenic induction for 7 and 14 days. Values are mean ± SD. * *p* < 0.05.

Figure 9A,B represents the images of ALP activity and ARS staining and corresponding quantitative analysis results. The ALP activity in p-Mg, Mg-1Zn-1Sn and Mg-1Zn-1Sn-0.2Sr groups was significantly enhanced. Quantitative analysis also showed that the ALP activities of p-Mg, Mg-1Zn-1Sn and Mg-1Zn-1Sn-0.2Sr groups were 1.8, 2.0 and 2.7 times that of the control group, respectively. Similarly, the ARS staining area of p-Mg, Mg-1Zn-1Sn and Mg-1Zn-1Sn-0.2Sr groups were significantly improved, and the absorbance of the extracellular matrix mineralization was 0.42 ± 0.06, 0.45 ± 0.03 and 0.60 ± 0.03, respectively, which were all significantly higher than that of the control group (0.25 ± 0.01). These results indicated that the Mg-1Zn-1Sn-0.2Sr group has the strongest osteoinductive activity. Figure 9C shows the expression of COL-I protein in cells after being cultured in various extracts for 14 days. Obviously, the Mg-1Zn-1Sn-0.2Sr group had the strongest fluorescence intensity among all groups, the order of fluorescence intensity was Mg-1Zn-1Sn-0.2Sr > Mg-1Zn-1Sn > p-Mg > Control. Our experimental results on COL-I expression were consistent with previous studies, which demonstrated that strontium has the ability to enhance the synthesis of COL-I [38,39].

We further explored the effect of various extracts on osteogenic differentiation of MC3T3-E1 cells on the molecular level. Runx2 is identified as a key transcription factor at the early stage of bone development [40]. OPN, a negatively charged non-collagenous bone matrix glycoprotein, is closely related to the formation of the bone matrix. Bglap is a vitamin K-dependent calcium-binding protein synthesized and secreted by osteoblasts, which is associated with the maturation of osteoblasts and matrix mineralization [41]. As shown in Figure 9D, the expression of these three genes in p-Mg, Mg-1Zn-1Sn and Mg-1Zn-1Sn-0.2Sr groups was significantly higher than the control group. In accordance with the previous results, the expression of the aforementioned genes related to osteogenesis was much stronger in the Mg-1Zn-1Sn-0.2Sr group than the other groups, both at day 7 and day 14. Sr has received widespread attention because of its not only stimulating osteoblast differentiation but also inhibiting osteoclast differentiation [42]. Specifically, Sr has the function of promoting the proliferation of osteoblasts by regulating calcium-sensing receptors and phosphorylation of extracellular regulated protein kinases1/2, as well as inhibiting bone resorption by reducing receptor activator of NF-κB ligand (RANKL) or enhancing the expression of osteoprotegerin [43]. In addition, Sr can also positively affect the interaction between osteocytes and osteoblasts by regulating the paracrine signal transduction [44].

Recently, Geoffroy et al. [45] have confirmed that Sr is related to the regulation of selective osteoinductive genes/their induction products. In this study, though the concentration of Sr is lower than 1.75 μg/mL (the lower limit strontium ranelate stimulates the proliferation and differentiation of osteoblasts) [46,47], the improvement of trace Sr in osteogenic activity of Mg-1Zn-1Sn-based alloy cannot be ignored. Park et al. [48] also reported that Sr ion concentrations as low as 103–135 ng/mL can still enhance osteogenic differentiation. We infer that the co-release of various metal ions such as Mg, Zn, Sn and Sr during the material degradation may have a synergistic effect, which greatly decreases the limit value, but the specific mechanism needs further study.

## 4. Conclusions

In this work, the possibility of using Mg-1Zn-1Sn and Mg-1Zn-1Sn-0.2Sr alloys as biomedical materials was explored for the first time; we systematically investigated their microstructure, surface characteristics, mechanical properties, bio-corrosion behaviors, biocompatibility and biological activity. The major conclusions are as follows:

(1) Mg-1Zn-1Sn and Mg-1Zn-1Sn-0.2Sr alloys have excellent corrosion resistance (0.31 ± 0.05 and 0.20 ± 0.03 mm/y), low H_2_ evolution (0.42 ± 0.02 and 0.13 ± 0.01 mL·cm^−2^) and suitable mechanical strength (229 ± 1 and 245 ± 8 MPa) as well as better biocompatibility compared with p-Mg, showing their significant application potential for using as orthopedic implants.

(2) Mg-1Zn-1Sn and Mg-1Zn-1Sn-0.2Sr alloys have osteoinductive activity comparable to or even significantly better than p-Mg, which may benefit from the contribution of Sr.

(3) By incorporating 0.2 wt.% of Sr into the Mg-1Zn-1Sn-based alloy, the corrosion resistance, mechanical properties, biocompatibility and biological activity of the material are all enhanced, demonstrating that Sr is an ideal alloy element for Mg-1Zn-1Sn-based alloys.

## Figures and Tables

**Table 1:** Actual composition of the Mg-1Zn-1Sn and Mg-1Zn-1Sn-0.2Sr alloys (wt.%).

Nominal Composition	Actual Composition						
	Zn	Sn	Sr	Fe	Si	Ni	Mg
Mg-1Zn-1Sn	1.04	1.13	0	<0.01	<0.01	<0.001	Balance
Mg-1Zn-1Sn-0.2Sr	1.02	1.12	0.21	<0.01	<0.01	<0.001	Balance

**Table 2:** The chemical compositions of SBF.

Components	Concentration
NaCl	7.996 g/L
KCl	0.224 g/L
CaCl_2_	0.278 g/L
NaHCO_3_	0.350 g/L
MgCl·6H_2_O	0.305 g/L
K_2_HPO_4_·3H_2_O	0.228 g/L
Na_2_SO_4_	0.071 g/L
HCl (1 mol/L)	40 mL
(CH_2_OH)_2_CNH_2_	6.051 g/L

**Table 3:** Primer sequences used for qRT-PCR.

Gene	Primers (F = Forward; R = Reverse)
Runx2	F: 5′-ATCCAGCCACCTTCACTTACAAA-3′
	R: 5′-GGGACCATTGGGAACTGATAGG-3′
OPN	F: 5′-CCAAGCGTGGAAACACACAGCC-3′
	R: 5′-GGCTTTGGAACTCGCCTGACTG-3′
Bglap	F: 5′-GAGCTGCCCTGCACTGGGTG-3′
	R: 5′-TGGCCCCAGACCTCTTCCCG-3′
β-actin	F: 5′-ATCGTGGGCCGCCCTAGGCA-3′
	R: 5′-TGGCCTTAGGGTTCAGAGGGG-3′

**Table 4:** The average corrosion rate calculated from H_2_ evolution, mass loss and I_corr_ of p-Mg, Mg-1Zn-1Sn and Mg-1Zn-1Sn-0.2Sr alloys in SBF solution.

Materials	Corrosion Rate (mm/y) Calculated by H_2_	Corrosion Rate (mm/y) Calculated by Mass Loss	Corrosion Rate (mm/y) Calculated by I_corr_
Mg	0.72 ± 0.28	0.44 ± 0.09	0.61 ± 0.16
Mg-1Zn-1Sn	0.24 ± 0.09	0.31 ± 0.05	0.29 ± 0.08
Mg-1Zn-1Sn-0.2Sr	0.18 ± 0.02	0.20 ± 0.03	0.23 ± 0.05

## Data Availability

The data presented in this study are available on request from the corresponding author.
